# Peer review of "Zinc-Based Metal-Organic Frameworks in Drug Delivery, Cell Imaging, and Sensing"

_molecules, 2021, doi:10.3390/molecules27010100_

Round 1

Reviewer 1 Report

The review has reviewed the recent advances of the MOFs that their metal is Zn. The authors have focused on some critical bio-based applications of Zn-based MOFs and the synthesis of the MOFs. However, they could present the synthesis part better since there are many recent methods for the synthesis of Zn-based MOFs and they should mention the morphology engineering of the MOFs with templating approach such as what is published recently in Chemical Society Reviews about the "Hard-Templated Synthesis of MOFs for Advanced Applications" in the year 2021. 

-Furthermore, some other parts are too short and do not cover the state-of-art works.

-I recommend the author to list all Zn-MOFs with showing their building blocks.

-Some figures have less quality and cannot be published in the current format.

-The English should be revised and polished.

-Since the stability of the Zn-MOF is low, the authors can mention this in a sub-section or in the conclusion somewhere. 

Author Response

The review has reviewed the recent advances of the MOFs that their metal is Zn. The authors have focused on some critical bio-based applications of Zn-based MOFs and the synthesis of the MOFs. However, they could present the synthesis part better since there are many recent methods for the synthesis of Zn-based MOFs and they should mention the morphology engineering of the MOFs with templating approach such as what is published recently in Chemical Society Reviews about the "Hard-Templated Synthesis of MOFs for Advanced Applications" in the year 2021.

Response: Thanks for the reviewer’s kind suggestion, more description for the synthesis of Zn-based MOFs with templating approach has been added in the revised manuscript. Please see the revised part 2.5 highlighted with red marks in the revised manuscript.

-Furthermore, some other parts are too short and do not cover the state-of-art works.

Response: According to the reviewer’s advice, related data have been added in the revised manuscript, such as part 3.1.

-I recommend the author to list all Zn-MOFs with showing their building blocks.

Response: Thank the reviewer’s good advice, a list showing MOFs with their building blocks in the form of Table 1 has been added in the revised manuscript.

-Some figures have less quality and cannot be published in the current format.

Response: According to the referee’s advice, quality of Figures have been improved to 300 dpi as per journal’s requirement.

-The English should be revised and polished.

Response: We are very sorry for these errors, we have carefully further reviewed and revised the English to address spelling, grammar mistakes.

-Since the stability of the Zn-MOF is low, the authors can mention this in a sub-section or in the conclusion somewhere.

Response: According to the referee’s suggestion, more description for stability of Zn-MOF has been added in a form of Table 2.

Reviewer 2 Report

Review of manuscript “Zinc-Based Metal-Organic Frameworks in Drug Delivery, Cell Imaging, and Sensing” submitted to Molecules.

This manuscript attempts to review the current standing in research fields of drug delivery, biomedical imaging and chemo-sensing using zinc metal-organic frameworks, Zn-MOFs.

However, large parts of this Review manuscript are taken not from original research publications by others, but from other reviews on the related topics, with slight rewording. Examples are provided below. The Review article is not really intended to be the compilation of other Reviews, but a critical analysis of original research papers in the field.

Further, there are existing reviews on related topics and this work has little, if any, value to the reader. In addition, the selection of topics to review is not well justified.

Many citations are referring to irrelevant papers.

Next, the English grammar is very poor in many sentences making this manuscript very hard to read and understand. Authors are highly encouraged to seek help of professional proof-reader and editor of English grammar.

Also, Figures from the cited articles are not provided the necessary copying permissions. This potentially constitutes copyright infringement and legal consequences.

In this form, this manuscript is not acceptable to any journal. I suggest rejecting this manuscript.

Major deficiencies

1) The lack of novelty in reviewing this research field. The already published reviews cover most of the material.

For example, see the review of 2019, https://doi.org/10.1080/03602532.2019.1632887

“Zinc-based metal–organic frameworks as nontoxic and biodegradable platforms for biomedical applications: review study”

which has covered biomedical studies reviewed in this manuscript.

2) Inconsistent selection of topics to review

Title of this manuscript “Zinc-Based Metal-Organic Frameworks in Drug Delivery, Cell Imaging, and Sensing” lacks consistency.

“Drug Delivery” and “Cell Imaging” belong to biomedical field, while “Sensing” does not.

Did authors perhaps discuss sensing related to biomedical field? This is not the case as sub-chapter “4.4. Chemosensors composed of Zn-based MOFs” discusses various toxic chemicals and not chemicals involved into biomedical processes.

3) The English grammar is very poor in quite many sentences. This makes the manuscript hard or impossible to understand. Few of many instances:

Page 3, lines 98-99:

“As the ultrasonic technique has proved more effective in fabricating Zn-based MOFs with fluorescent characteristics mainly employed for ethylene amine sensing.”

While it looks to be the compound sentence, the second noun is missing.

Page 4, lines 131-132:

“However, first recorded the microwave-assisted production of CPM-5 in 2012 [34].”

The noun is missing; who has recorded something in 2012?

Page 5, lines 141-142:

“Biological friendly composition of metal-organic frameworks has enabled their application in biomedical, however…”

The word “biomedical” is an adjective. Did authors mean “in biomedical field”?

4) The excessive reuse of content from other reviews, often of no relevance to the topic of this manuscript.

See page 3, lines 91-95 of the manuscript:

“In HKUST-1, Cu paddlewheel structural building units (SBUs) are coordinated to form three-dimensional porous cubic networks through 1,3,5-92 benzenetricarboxylate (BTC). On the other hand, Zn4O (DMF)8(C6H5Cl), where BDC accounts for the terephthalic acid and the DMF for dimethyl formamide, comprised of MOF-5, Zn4O(BDC)3·(DMF)8(C6H5Cl), clusters related to linear BDC linkers [25].”

Compare this with text in the other review paper, Sci. Technol. Adv. Mater. 16 (2015) 054202, page 2 herein:

“In HKUST-1, Cu paddlewheel secondary building units (SBUs) are coordinated via 1,3,5-benzenetricarboxylate (BTC) to form three dimensional porous cubic networks (figure 1). On the other hand, MOF-5, with the chemical formula of Zn4O (BDC)3·(DMF)8(C6H5Cl) where BDC stands for terephthalic acid and DMF for dimethylformamide, consists of Zn4O clusters.”

Interestingly, HKUST-1 described here is copper MOF and not zinc MOF, and therefore its discussion is outside of scope of this Review manuscript.

See page 5, lines 154-155:

“Furthermore, a solid grinding technique was used to prepare Au-MOF composites by grinding the Au particles with MIL-53(Al), MOF-5, and HKUST-11 [39].”

The reader goes to reference [39] to find the information from the research paper about “a solid grinding technique… to prepare Au-MOF composites”. But in fact, reference [39] is a review of 30 pages long about many items:

Lin, W.; Hu, Q.; Jiang, K.; Cui, Y.; Yang, Y.; Qian, G. A porous Zn-based metal- Liu, J.; Huang, J.; Zhang, L.; Lei, J. Multifunctional 689 metal–organic framework heterostructures for enhanced cancer therapy. Chem. Soc. Rev. 2021, 50, 1188-1218.

Instead, the reader needs the citation of specific research paper about Au-MOF composites.

See page 10, Figure 6.

Per citation [48], this Figure is from paper

Liu, W., Y. Pan, W. Xiao, H. Xu, D. Liu, F. Ren, X. Peng and J. Liu, 2019. Recent developments on zinc (ii) metal–organic frame-710 work nanocarriers for physiological ph-responsive drug delivery. MedChemComm, 10(12): 2038-2051.

However, the cited paper [48] is itself a review of research papers by others. At the end, this reviewer is unable to find to which, specifically, research paper Figure 6 belong? The review manuscript by authors written in such a way has no value to the reader.

5) In many cases, the citation numbers [x] given by authors in the text simply do not match the actual content of what they cite. This is in addition to poor understanding of the science in the cited papers. Just a few examples.

Example #1:

Page 6, lines 179-180:

“release potential (64.9 and 81.9 %) for 5-fluorouracil, constituting a negative load capacity (around 53.3 % wt.) [8].”

First, “a negative load capacity” is scientifically impossible. “Load” is the amount of the compound added to the other compound, while “capacity” is the maximum amount of the compound which can be added to the other compound. Of course, none of these can be negative.

Second, the cited reference [8] is:

Maddigan, N.K.; Tarzia, A.; Huang, D. M.; Sumby, C. J.; Bell, S .G.; Falcaro, P.; Doonan, C.J. Protein surface functionalisation as 625 a general strategy for facilitating biomimetic mineralisation of zif-8. Chem. Sci. 2018, 9, 4217-4223

and it has nothing to do with 5-fluorouracil.

Example #2:

Page 2, line 73 and page 3, lines 74-75:

“Using these approaches to make MOFs allows you to tune their structure and operation throughout the fabrication process, where visitor compounds can connect to vacant spaces and react with them [18].”

Reference [18] cited by authors is:

Park, M.; Kim, J. Y.; Son, H. J.; Lee, C. H.; Jang, S. S.; Ko, M. J. Low temperature solution-processed Li-doped SnO2 as an effective electron transporting layer for high-performance flexible and wearable perovskite solar cells. Nano Energy 2016, 26, 645 208–215.

and it does not describe any MOF.

6) Excessive use of unprofessional terminology and incorrect scientific statements.

When someone writes a Review paper for others in the field, at least the language must be consistent with what is used by professionals in the field and is written in the textbooks. Often, this is not the case in this manuscript.

One example with many such errors. Page 9, lines 273-276:

“The fluorescent pictures of the NPs interacted with HeLa cells significantly stronger than those of HEK 293T cells”…

“The fluorescent pictures” cannot interact with “HeLa cells”. Bad English completely distorts understanding of this sentence.

In the same sentence further:

“and fluorescence microscopy demonstrated a 6-fold enhancement in the absorption spectra of the HeLa cells…”

Scientifically, fluorescence microscopy cannot demonstrate an enhancement in the absorption spectra. Instead, it could demonstrate an enhancement in the fluorescence spectra.

And finally in the same sentence:

“demonstrating that somehow this approach possesses outstanding targeting efficacy.”

This reviewer believes that colloquial language such as “somehow” is inappropriate in scholastic paper.

Another example. Page 9, lines 306-307:

“which also has excellent compatibility of cells and pH-responsive drug-emission action.”

There is no such a term as “drug emission” in the literature to describe neither of those. Did authors mean “drug release” or “drug delivery”?

Yet another example. Page 9, lines 320-322:

“Because the physical relationship involving adenosine triphosphate (ATP) and Zn2+ is much stronger than both the imidazole and Zn2+…”

There is no such thing as “physical relationship” in Chemistry. Did authors want to say “chemical bond” or “interaction”?

Another example. Page 10, lines 348-349:

“Designing pH-dependent medicinal carriers in Zn-based MOFs for medical applications is a fantastic achievement.”

There is nothing “fantastic” here, the chemistry behind is very simple, and the word “fantastic” is not appropriate for scholastic literature. In fact, the pH dependent release of drugs from zinc MOFs is known since at least 2013: see paper “pH-induced different crystalline behaviors in extended metal–organic frameworks based on the same reactants”, Dalton Trans., 2013, 42, 6294–6297.

7) In this review, most Figures (Figures 2-9) are copied from papers by others. While the citing references are provided, none of the copied Figures has Copyright Permission. This is a violation of the copyright law. If authors contemplate resubmission of this manuscript elsewhere, authors must obtain permissions of publishers to reuse the copied Figures (except Figure 1) and provide the proper license numbers of copyright permissions.

Author Response

Response to the Reviewer 2:

This manuscript attempts to review the current standing in research fields of drug delivery, biomedical imaging and chemo-sensing using zinc metal-organic frameworks, Zn-MOFs.

However, large parts of this Review manuscript are taken not from original research publications by others, but from other reviews on the related topics, with slight rewording. Examples are provided below. The Review article is not really intended to be the compilation of other Reviews, but a critical analysis of original research papers in the field. Further, there are existing reviews on related topics and this work has little, if any, value to the reader. In addition, the selection of topics to review is not well justified.

Response: We are very sorry for these errors; we have carefully further reviewed and removed the irrelevant papers and added more data from different papers.

Many citations are referring to irrelevant papers.

Response: We are very sorry for these errors; we have carefully further reviewed these errors and removed those irrelevant papers.

Next, the English grammar is very poor in many sentences making this manuscript very hard to read and understand. Authors are highly encouraged to seek help of professional proof-reader and editor of English grammar.

Response: We are very sorry for this trouble, we have carefully further reviewed and revised the English to address spelling and grammar errors.

Also, Figures from the cited articles are not provided the necessary copying permissions. This potentially constitutes copyright infringement and legal consequences.

Response: Thank the review for your attention on this issue, copyright permissions will be provided with the revised manuscript.

Major deficiencies

  • The lack of novelty in reviewing this research field. The already published reviews cover most of the material.

“Zinc-based metal–organic frameworks as nontoxic and biodegradable platforms for biomedical applications: review study” which has covered biomedical studies reviewed in this manuscript.

Response: We are very sorry for these errors and we have carefully further reviewed and added more data from different papers for the novelty purpose. This review is aimed at providing the recent progress on the applications of different Zn based MOFs. Moreover, the review shows in details the toxicity and stability as well as effects of pH on the application of MOFs. In addition, we also discuss the effectiveness of different Zn based MOFs for drug delivery and cell imaging. Furthermore, we provide the challenges and evidence gaps that encounter the use of different linkers to make the application of MOF more effective.

2) Inconsistent selection of topics to review

Title of this manuscript “Zinc-Based Metal-Organic Frameworks in Drug Delivery, Cell Imaging, and Sensing” lacks consistency.

“Drug Delivery” and “Cell Imaging” belong to biomedical field, while “Sensing” does not.

Did authors perhaps discuss sensing related to biomedical field? This is not the case as sub-chapter “4.4. Chemosensors composed of Zn-based MOFs” discusses various toxic chemicals and not chemicals involved into biomedical processes.

Response: According to the reviewer’s suggestion, the structure of this part has been reorganized.

3) The English grammar is very poor in quite many sentences. This makes the manuscript hard or impossible to understand. Few of many instances:

Response: We are very sorry for these errors, we have carefully further reviewed and revised the English to address spelling and grammar.

Page 3, lines 98-99:

“As the ultrasonic technique has proved more effective in fabricating Zn-based MOFs with fluorescent characteristics mainly employed for ethylene amine sensing.”

While it looks to be the compound sentence, the second noun is missing.

Response: We are very sorry for this error, we have carefully further reviewed and revised it.

“As the ultrasonic technique is more effective in fabricating Zn-based MOFs with fluorescent characteristics, mainly for the accurate sensing of ethylene amine.”

Page 4, lines 131-132:

“However, first recorded the microwave-assisted production of CPM-5 in 2012 [34].”

The noun is missing; who has recorded something in 2012?

Response: We are very sorry for this error, we have carefully further reviewed and revised it.

Page 5, lines 141-142:

“Biological friendly composition of metal-organic frameworks has enabled their application in biomedical, however…”

The word “biomedical” is an adjective. Did authors mean “in biomedical field”?

Response: Thanks the reviewer and we are very sorry for these errors, we have carefully further reviewed and revised the sentence to fix it.

This sentence has been changed as: “Biological friendly composition of metal-organic frameworks has enabled their application in biomedical and environmental fields.”

4) The excessive reuse of content from other reviews, often of no relevance to the topic of this manuscript.

Response: We are very sorry for these errors, we have carefully further reviewed and removed the irrelevant data and added more data from relevant papers.

See page 3, lines 91-95 of the manuscript:

“In HKUST-1, Cu paddlewheel structural building units (SBUs) are coordinated to form three-dimensional porous cubic networks through 1,3,5-92 benzenetricarboxylate (BTC). On the other hand, Zn4O (DMF)8(C6H5Cl), where BDC accounts for the terephthalic acid and the DMF for dimethyl formamide, comprised of MOF-5, Zn4O(BDC)3·(DMF)8(C6H5Cl), clusters related to linear BDC linkers [25].

Compare this with text in the other review paper, Sci. Technol. Adv. Mater. 16 (2015) 054202, page 2 herein:

“In HKUST-1, Cu paddlewheel secondary building units (SBUs) are coordinated via 1,3,5-benzenetricarboxylate (BTC) to form three dimensional porous cubic networks (figure 1). On the other hand, MOF-5, with the chemical formula of Zn4O (BDC)3·(DMF)8(C6H5Cl) where BDC stands for terephthalic acid and DMF for dimethylformamide, consists of Zn4O clusters.”

Interestingly, HKUST-1 described here is copper MOF and not zinc MOF, and therefore its discussion is outside of scope of this Review manuscript.

Response: We are very sorry for these errors, we have carefully further reviewed and replaced the irrelevant data with relevant one.

See page 5, lines 154-155:

“Furthermore, a solid grinding technique was used to prepare Au-MOF composites by grinding the Au particles with MIL-53(Al), MOF-5, and HKUST-11 [39].”

The reader goes to reference [39] to find the information from the research paper about “a solid grinding technique… to prepare Au-MOF composites”. But in fact, reference [39] is a review of 30 pages long about many items:

Lin, W.; Hu, Q.; Jiang, K.; Cui, Y.; Yang, Y.; Qian, G. A porous Zn-based metal- Liu, J.; Huang, J.; Zhang, L.; Lei, J. Multifunctional 689 metal–organic framework heterostructures for enhanced cancer therapy. Chem. Soc. Rev. 2021, 50, 1188-1218.

Instead, the reader needs the citation of specific research paper about Au-MOF composites.

Response: We are very sorry for these errors, we have carefully further reviewed and replaced the irrelevant data with relevant one.

See page 10, Figure 6.

Per citation [48], this Figure is from paper

Liu, W., Y. Pan, W. Xiao, H. Xu, D. Liu, F. Ren, X. Peng and J. Liu, 2019. Recent developments on zinc (ii) metal–organic frame-710 work nanocarriers for physiological ph-responsive drug delivery. MedChemComm, 10(12): 2038-2051.

However, the cited paper [48] is itself a review of research papers by others. At the end, this reviewer is unable to find to which, specifically, research paper Figure 6 belong? The review manuscript by authors written in such a way has no value to the reader.

Response: Thank the reviewer for the good advice, we have removed that figure for making it more valuable for the readers

5) In many cases, the citation numbers [x] given by authors in the text simply do not match the actual content of what they cite. This is in addition to poor understanding of the science in the cited papers. Just a few examples.

Example #1:

Page 6, lines 179-180:

“release potential (64.9 and 81.9 %) for 5-fluorouracil, constituting a negative load capacity (around 53.3 % wt.) [8].”

First, “a negative load capacity” is scientifically impossible. “Load” is the amount of the compound added to the other compound, while “capacity” is the maximum amount of the compound which can be added to the other compound. Of course, none of these can be negative.

Second, the cited reference [8] is:

Maddigan, N.K.; Tarzia, A.; Huang, D. M.; Sumby, C. J.; Bell, S .G.; Falcaro, P.; Doonan, C.J. Protein surface functionalisation as 625 a general strategy for facilitating biomimetic mineralisation of zif-8. Chem. Sci. 2018, 9, 4217-4223 and it has nothing to do with 5-fluorouracil.

Response: We are very sorry for these errors, we have carefully further reviewed and revised.

Example #2:

Page 2, line 73 and page 3, lines 74-75:

“Using these approaches to make MOFs allows you to tune their structure and operation throughout the fabrication process, where visitor compounds can connect to vacant spaces and react with them [18].”

Reference [18] cited by authors is:

Park, M.; Kim, J. Y.; Son, H. J.; Lee, C. H.; Jang, S. S.; Ko, M. J. Low temperature solution-processed Li-doped SnO2 as an effective electron transporting layer for high-performance flexible and wearable perovskite solar cells. Nano Energy 2016, 26, 645 208–215. and it does not describe any MOF.

Response: We are very sorry for these errors, we have carefully further reviewed and replace the reference accordingly.

6) Excessive use of unprofessional terminology and incorrect scientific statements.

When someone writes a Review paper for others in the field, at least the language must be consistent with what is used by professionals in the field and is written in the textbooks. Often, this is not the case in this manuscript.

One example with many such errors. Page 9, lines 273-276:

“The fluorescent pictures of the NPs interacted with HeLa cells significantly stronger than those of HEK 293T cells”…

“The fluorescent pictures” cannot interact with “HeLa cells”. Bad English completely distorts understanding of this sentence.

In the same sentence further:

“and fluorescence microscopy demonstrated a 6-fold enhancement in the absorption spectra of the HeLa cells…”

Scientifically, fluorescence microscopy cannot demonstrate an enhancement in the absorption spectra. Instead, it could demonstrate an enhancement in the fluorescence spectra.

And finally in the same sentence: “demonstrating that somehow this approach possesses outstanding targeting efficacy.”

This reviewer believes that colloquial language such as “somehow” is inappropriate in scholastic paper.

Response: We are sorry for the language errors in the manuscript and totally agree with the reviewer suggestions. Language and choice of words is really important to make the manuscript understandable for the readers. We have carefully reviewed the language problems throughout the manuscript and made the corrections.

Another example. Page 9, lines 306-307:

“which also has excellent compatibility of cells and pH-responsive drug-emission action.”

There is no such a term as “drug emission” in the literature to describe neither of those. Did authors mean “drug release” or “drug delivery”?

Response: We are very sorry for these errors, we have carefully further reviewed and revised the error as “which also has excellent compatibility of cells and pH-responsive drug delivery”.

Yet another example. Page 9, lines 320-322:

“Because the physical relationship involving adenosine triphosphate (ATP) and Zn2+ is much stronger than both the imidazole and Zn2+…”

There is no such thing as “physical relationship” in Chemistry. Did authors want to say “chemical bond” or “interaction”?

 Response: We are very sorry for these errors, we have carefully further reviewed and revised this as

“Because the physical interaction between adenosine triphosphate (ATP) and Zn2+ is much stronger than both the imidazole and Zn2+…”

Another example. Page 10, lines 348-349:

“Designing pH-dependent medicinal carriers in Zn-based MOFs for medical applications is a fantastic achievement.”

There is nothing “fantastic” here, the chemistry behind is very simple, and the word “fantastic” is not appropriate for scholastic literature. In fact, the pH dependent release of drugs from zinc MOFs is known since at least 2013: see paper “pH-induced different crystalline behaviors in extended metal–organic frameworks based on the same reactants”, Dalton Trans., 2013, 42, 6294–6297.

Response: Thanks for your attention to this point. We have made some corrections in it.

“It's a remarkable achievement to design pH-dependent medicinal carriers in Zn-based MOFs for medical applications.”

7) In this review, most Figures (Figures 2-9) are copied from papers by others. While the citing references are provided, none of the copied Figures has Copyright Permission. This is a violation of the copyright law. If authors contemplate resubmission of this manuscript elsewhere, authors must obtain permissions of publishers to reuse the copied Figures (except Figure 1) and provide the proper license numbers of copyright permissions.

Response: Thank the review for your attention on this issue, copyright permissions will be provided with the revised manuscript.

Reviewer 3 Report

This work by Li and colleagues is a comprehensive review of the application of Zn-based MOFs in sensing, drug delivery and cell imaging.

There are some points that the authors should address in any subsequent revision:

  1. References 20, 45 and 49 need to be reformatted (some problems with blue words).
  2. There should be representative X-ray figures of some examples.
  3. The authors could consider to mention the use of zinc organic frameworks in the recognition of huge molecules (not only small ones), such as glycopeptides (https://doi.org/10.1021/acsami.6b08218), polymers (https://doi.org/10.1039/D1SC03770F, https://doi.org/10.1038/s41467-018-06099-z, https://doi.org/10.1002/anie.202102794) or fullerenes derivatives (https://doi.org/10.1038/nature02311, https://doi.org/10.1021/acs.chemmater.0c03796, https://doi.org/10.1002/anie.202100996).
  4. The authors should increase the content of section 4.4.

Author Response

Response to the Reviewer 3:

This work by Li and colleagues is a comprehensive review of the application of Zn-based MOFs in sensing, drug delivery and cell imaging. There are some points that the authors should address in any subsequent revision:

  1. References 20, 45 and 49 need to be reformatted (some problems with blue words).

Response: Thanks for your attention to this part. We have reformatted all the references on Endnote according to the journal guideline

  1. There should be representative X-ray figures of some examples.

Response: Thank to the reviewer for advice. We have added some X-ray figures in relevant parts.

  1. The authors could consider to mention the use of zinc organic frameworks in the recognition of huge molecules (not only small ones), such as glycopeptides (https://doi.org/10.1021/acsami.6b08218), polymers (https://doi.org/10.1039/D1SC03770F, https://doi.org/10.1038/s41467-018-06099-z, https://doi.org/10.1002/anie.202102794) or fullerenes derivatives (https://doi.org/10.1038/nature02311, https://doi.org/10.1021/acs.chemmater.0c03796, https://doi.org/10.1002/anie.202100996).

The authors should increase the content of section 4.4.

Response: Nice to hear this suggestion from the reviewer. We have revised and added more relevant data to increases the content in the revised section 5.4.

Round 2

Reviewer 1 Report

The authors have revised the manuscript accordingly and it is publishable in the current version.